# Effects of *Phellinus linteus* extract on immunity improvement: A CONSORT-randomized, double-blinded, placebo-controlled trial

**DOI:** 10.1097/MD.0000000000030226

**Published:** 2022-08-26

**Authors:** Yong Ho Ku, Jae Hui Kang, Hyun Lee

**Affiliations:** a Department of Acupuncture and Moxibustion Medicine, College of Korean Medicine, Daejeon University, Daejeon, Republic of Korea; b Department of Acupuncture and Moxibustion Medicine, Cheonan Korean Medicine Hospital of Daejeon University, Seobuk-gu, Republic of Korea.

**Keywords:** immune function, NK cell, *Phellinus linteus*, randomized controlled trial

## Abstract

Immunity protects the body from external threats and prevents the development of cancer. Biological response modifiers extracted from natural sources are being actively studied, and the immunostimulatory and anticancer effects of various types of fungi have been reported. However, there are no previous clinical studies on the immune-enhancing effect of *Phellinus linteus* (PL). Lactate dehydrogenase cytotoxicity assay is a prerequisite in order to get approval for using PL as a raw material in functional supplements and medicines in Korea. However, due to the absence of precedent clinical trials, the use of PL in supplements has been hindered. but there is no precedent clinical trial using it. We conducted a randomized, double-blinded, placebo-controlled trial to confirm the efficacy and safety of PL extract for the improvement of immunity using the lactate dehydrogenase cytotoxicity assay.

**Methods:**

A total of 98 subjects were enrolled and randomly assigned to 2 groups. Subjects in the PL and placebo groups received 1000 mg of PL extract and 1000 mg of dextrin per day, respectively (one capsule, twice every day for 8 weeks). The primary outcome measured was the activity of natural killer cells. Secondary outcomes were the levels of TNF-α, IFN-γ, IL-1β, IL-2, IL-6, IL-12, IgG1, IgG2, and IgM. Safety was evaluated using laboratory tests.

**Results:**

NK cell activity was significantly increased in the PL group compared to the placebo group (*P* < .05). Despite the absence of significant changes in secondary outcomes, there was a tendency for improvement in the PL group. PL extract-related adverse outcomes, particularly in liver and renal function, were not observed.

**Conclusion:**

PL extract may improve immunity and is safe to be consumed orally.

## 1. Introduction

Immunity protects the human body from pathogens and prevents the development of cancer. However, aging populations and modern lifestyles (i.e., westernized eating habits, stress, and lack of exercise) adversely impact the immune function of individuals.^[1]^ A weakened immunity impairs resistance to infection and is associated with the occurrence of various diseases. Therefore, immunomodulatory biomaterials derived from herbal medicines are used to reduce the side effects of immunosuppression and to enhance immunity.^[2]^

*Phellinus linteus* (PL) is a fungus that grows on mulberry trees. PL exhibits an anticancer and antioxidant effects,^[3]^ enhances humoral and cellular immunity,^[4]^ and activates interleukin-12 (IL-12), interferon-γ (IFN-γ), and B cell,^[5]^ and natural killer (NK) cell.^[6,7]^ Despite the cost and limited supply of the fruiting body of PL, it is widely used as one of the major ingredients for health supplements and medicines thanks to its pharmacological activity.^[8]^ However, there are no previous clinical trials investigating the effects of PL on immunity. The lactate dehydrogenase (LDH) cytotoxicity assay has been strongly recommended by the National Institute of Food and Drug Safety Evaluation of Korea to measure the NK cell activity, and this is mandatory to use PL extract in functional supplements. In general, either flow cytometry or enzyme-linked immunosorbent assay is used to measure NK cell activity clinically. Hence, the activity of NK cells was measured using both experimental methods to clinically evaluate the effects of PL mycelium extract on human immune system.

The immunostimulatory and antiinflammatory effects of fermented extract from the PL KCCM KSW01 strain have been reported in a nonclinical study.^[9]^ In the present clinical trial, fermented PL extract or placebo was administered to 98 patients for 8 weeks, and the effects of PL extract on immune activity were analyzed using the LDH cytotoxicity assay.

## 2. Materials and methods

### 2.1. Trial design and protocol

A randomized, double-blinded, placebo-controlled trial was designed to examine the efficacy and safety of PL extract in improving the immunity. This clinical trial was registered at the Korean National Clinical Research Information Service (CRIS-KCT0005460) and approved by the Institutional Review Board (IRB) at Daejeon University Cheonan Korean Medicine Hospital (DJUMC-2020-BM-07). The study protocol had been previously published.^[10]^ The study was performed according to the tenets of the Declaration of Helsinki and good clinical practice guidelines. The duration of this clinical trial was 8 weeks.

### 2.2. Objective and Sample calculation

NK cell activity (primary outcome) and the cytokine levels (secondary outcomes) were measured. The calculation of the sample size was based on the results of a pilot clinical trial. In the pilot study, the standard deviation for NK cell activity in the PL and placebo groups was 569.72 and 375.16, respectively, and the pooled standard deviation was 442.60. Based on this statistical index, the number of subjects required for a significance level of .05 (both sides) and a power of 80% was calculated. Using G*power 3.1.9.4, the calculated minimum number of subjects required to confirm statistical significance was 41 per group. Hence, taking into account a 15% dropout rate, this clinical trial aimed to recruit a total of 98 subjects (49 per group).

### 2.3. Inclusion and Exclusion criteria

The inclusion criteria were: age 20 to 65 years; white blood cell (WBC) count 3–10 × 10^3^/μL; diagnosis of upper respiratory tract infection or common cold within 1 year; and voluntary participation in the clinical trial.

The exclusion criteria were as follows: presence of acute or chronic cardiocerebrovascular, immune, respiratory, hepatobiliary, renal, urinary, nervous, musculoskeletal, psychotic, infectious, hematologic, or neoplastic disease; uncontrolled hypertension; uncontrolled diabetes; aspartate aminotransferase (glutamic oxaloacetic transaminase) and alanine aminotransferase (glutamic pyruvic transaminase) levels 3-fold higher than the upper limit; creatinine levels >2.4 mg/dL and >1.8 mg/dL in males and females, respectively; having received immune-related health supplements within 2 weeks prior to screening; presence of severe gastrointestinal symptoms; pregnancy, breastfeeding, or pregnancy plans during the study period; sensitivity or allergy to the PL extract; plan to participate in other studies during the study period; use of other research drugs within 4 weeks before the initiation of this study; and deemed inappropriate for participation in this study by the investigator due to any reason.

### 2.4. Randomization and Blinding

A total of 98 subjects were randomly assigned (1:1) to the PL (49 subjects) and placebo (49 subjects) groups using block randomization. The size of the block was randomly set by a statistician for blinding. The total number of random assignments was generated at approximately 120% of the target number of recruits. A 3-digit randomized number was assigned to the recruited subjects in sequence, and the PL extract or placebo was distributed to the subjects according to the randomization number. Randomization tables were generated by the statistical experts using SPSS Version 22.0 (SPSS Statistics for Windows Version 22.0; IBM Corp., Armonk, NY). The manufacturer received randomization tables and manufactured clinical trial capsules for the clinical trial. The sponsor sealed the random assignment details of each subject in a nontransmissive envelope and provided them to the hospital. Investigator and subjects were blinded with blinding procedure.

### 2.5. Intervention

Subjects in the PL and placebo groups received 1000 mg/d of PL extract and 1000 mg/d of dextrin, respectively (500 mg capsules were taken twice every day at fixed intervals). The capsules were produced by Hankookshinyak Pharmaceutical (Nonsan, Republic of Korea) and did not exhibit any appearance-wise or size-wise difference. The total duration of the clinical trial was 8 weeks. The test drug was administered according to the protocol. The PL extract and placebo were distributed at Visits 1 and 2, and the subjects were evaluated at Visits 1 and 3 according to the schedule.

### 2.6. Outcomes

The primary outcome was NK cell activity. The secondary outcomes were WBC, and the levels of tumor necrosis factor-α (TNF-α), IFN-γ, IL-1β, IL-2, IL-6, IL-12, immunoglobulin (Ig) G1, IgG2, and IgM. The outcomes were measured before (Visit 1) and after (Visit 3) administration, and the degree of change was analyzed.

Blood samples were collected to analyze the levels of AST, ALT, ALP, γ-GTP, Total cholesterol, FBS, Total bilirubin, BUN, Creatinine, ESR, WBC, RBC, Hb, Hct, platelet, Triglyceride, Na, K, and Cl at Daejeon University Cheonan Korean Medicine Hospital. Blood samples were stored at room temperature immediately after blood collection, and the NK cell activity was analyzed at a concentration of 50:1, 25:1, 12.5:1 (the effector cell:target cell) to confirm the accuracy and the precision of the NK cell activity using Lymphoprep™ (STEMCELL Technologies, Vancouver, Canada), MojoSort™ Human NK Cell Isolation Kit (BioLegend, San Diego, United States) and CytoTox96 Non-Radioactive Cytotoxicty Assay (Promega, Madison, United States) at U2Bio Co., Ltd. (Seoul, Korea) on the day of blood collection. Immediately after blood collection, blood samples were stored at −20°C in the diagnostic laboratory at Daejeon University Cheonan Korean Medicine Hospital until the last blood collection of the last subjects was completed. All blood samples were transferred to GCCL Co., Ltd. (Yongin, Korea) to analyze the expression levels of TNF-α, IFN-γ, IL-1β, IL-2, IL-6, IL-12 using TNF-α HS M_Multiplex (Millipore, Burlington, United States), IFN-γ HS M_Multiplex (Millipore), IL-1β HS M_Multiplex (Millipore), IL-2 HS M_Multiplex (Millipore), IL-6 HS M_Multiplex, and IL-12 (p70) HS M_Multiplex (Millipore). Blood samples were transferred immediately to GC cell Co., Ltd. right after collection to analyze IgG1, IgG2, IgM using Human IgG and IgG subclass liquid reagent kits for use on the SPA plus (The Binding site, Birmingham, United Kingdom) and Tina-quant IgM Gen.2 (Roche, Basel, Switzerland). Urine human chorionic gonadotropin (HCG) tests were performed only during the screening process, with the exception of men and postmenopausal women.

### 2.7. Data analysis

The full analysis set (FAS) population was the main statistical analysis group. An analysis of the per protocol (PP) population was also conducted as a reference. All statistical analyses were performed using SPSS Statistics for Windows Version 22.0 (IBM Corp.), using a significance level of <.05 for both sides. Demographic variables measured during screening were analyzed for comparison between groups. Continuous data were analyzed using the *t* test. Categorical data were analyzed using the chi-squared test and Fisher exact test. Continuous variables between groups with normal distributions were analyzed using the independent *t* test for the efficacy of PL extract. A paired *t* test was performed if there was the data normality to analyze differences in a group over time. The Shapiro–Wilk test was used to test the normality of continuous variables. For the FAS analysis group, the missing values were replaced using the Last Observation Carried Forward method.

### 2.8. Withdrawal and Dropout

Subjects who received the test drugs for 8 weeks were considered to have completed the clinical trial. The subjects with the following criteria were excluded: withdrawal of consent; occurrence of a serious adverse event; violation of protocol; loss of contact; use of prohibited supplements or drugs; pregnancy; and investigator’s judgment. In case of dropout, the case report form was completed in detail, and the laboratory tests were performed to evaluate safety. Adverse events were tracked and observed until the cause was identified. In cases of serious events, we notified the IRB immediately.

## 3. Results

**Figure 1.**
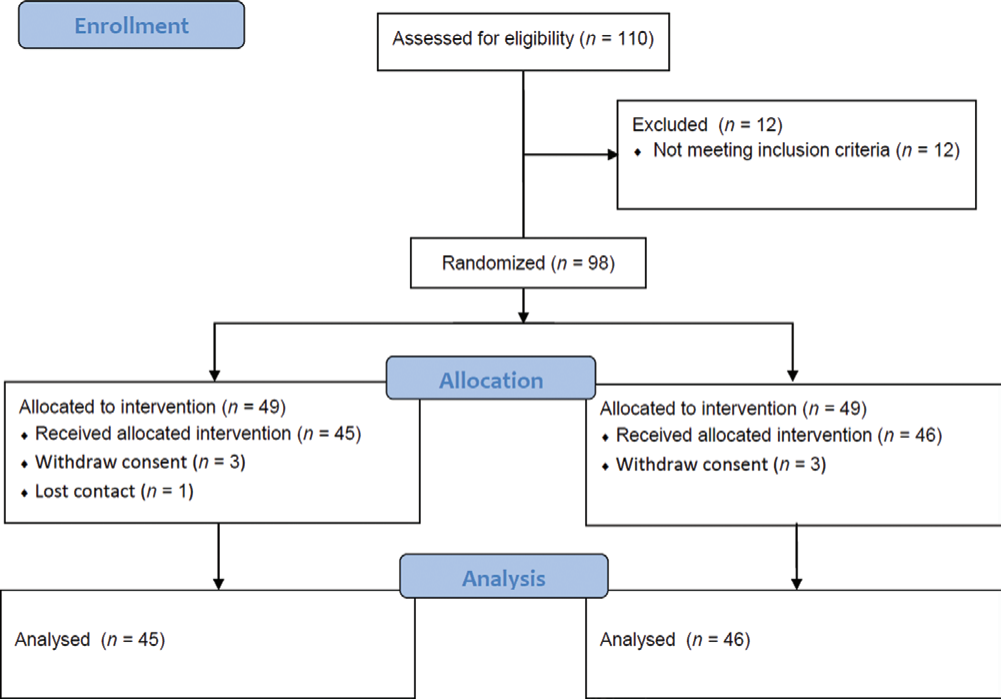
CONSORT 2010 flow diagram. Randomized placebo-controlled trial flow chart illustrates the study design and participant flow. CONSORT = consolidated standards of reporting trials.

From October 21, 2020 to March 4, 2021, recruitment and the follow-up of 91 subjects were implemented. The FAS analysis group consisted of all subjects for whom the data were obtained at least at one of the visits. The FAS population consisted of 91 subjects (PL group: n = 45; placebo group: n = 46), and the PP population consisted of 84 subjects (PL group: n = 41; placebo group: n = 43), excluding the 6 subjects (PL group: n = 3; placebo group: n = 3) who failed to undergo evaluation at Visit 3 due to blood collection failure and 1 subject in the PL group with <80% compliance (Fig. 1). The basic characteristics of the subjects were analyzed (Table 1). Both the FAS and PP populations did not show statistically significant differences in sex, age, weight, height, etc. The homogeneity of the 2 populations was recognized, and the randomized assignments were statistically appropriate.

**Table 1:** Baseline characteristics of the subjects.

Variable	FAS population			PP population		
	Placebo group (n = 46)	PL group (n = 45)	*P* value	Placebo group (n = 43)	PL group (n = 41)	*P* value
Sex						
Male	10 (21.7%)	7 (15.6%)	.449*	9 (20.9%)	7 (17.1%)	.653*
Female	36 (78.3%)	38 (84.4%)		34 (79.1%)	34 (82.9%)	
Drinking history						
No drinking	31 (67.4%)	33 (73.3%)	.731**	29 (67.4%)	30 (73.2%)	.811**
Moderate drinking	14 (30.4%)	12 (26.7%)		13 (30.2%)	11 (26.8%)	
Heavy drinking	1 (2.2%)	0 (0.0%)		1 (2.3%)	0 (0.0%)	
Smoking history						
No smoking	38 (82.6%)	38 (84.4%)	.833**	36 (83.7%)	34 (82.9%)	.626**
Smoking in the past	4 (8.7%)	2 (4.4%)		4 (9.3%)	2 (4.9%)	
Currently smoking	4 (8.7%)	5 (11.1%)		3 (7.0%)	5 (12.2%)	
Age (yr)	47.20 ± 12.70	46.02 ± 11.67	.648***	47.00 ± 13.11	46.71 ± 11.94	.915***
Weight (kg)	61.74 ± 9.78	59.18 ± 10.31	.227***	61.64 ± 10.06	59.75 ± 10.32	.400***
Height (cm)	161.36 ± 6.40	159.95 ± 5.87	.277***	161.43 ± 6.30	160.07 ± 6.09	.316**^*^

FAS = full analysis set, PL = *Phellinus linteus*, PP = per protocol.

* *P* values were derived from the chi-squared test.

** *P* values were derived from Fisher exact test.

*** *P* values were derived from the independent *t* test.

**Figure 2.**
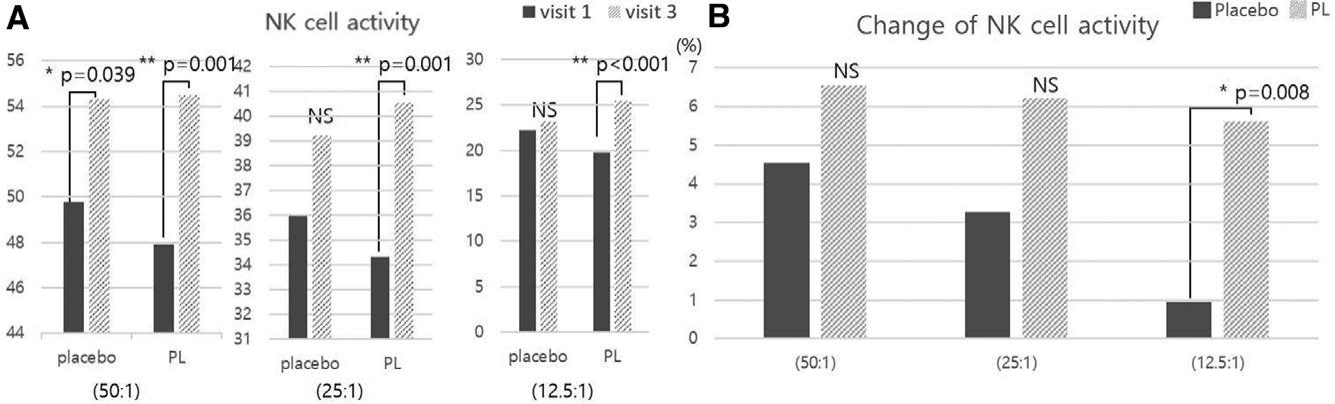
NK cell activity. (A) NK cell activity. (B) Change of NK cell activity. NK cell activity was increased in all cases. In particular, there was a significant increase in the PL group compared to the placebo at 12.5:1. NK = natural killer, NS = no significant.

**Figure 3.**
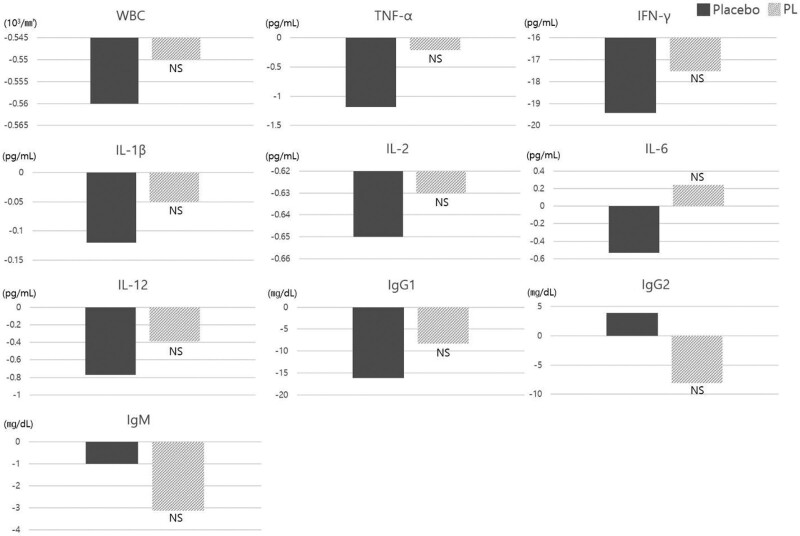
Changes in secondary outcomes. Secondary outcomes showed no significant difference. Changes in the levels of IgG2 and IgM were observed, but were not statistically significant. IL-1β = interleukin-1β, IL-2 = interleukin-2, IL-6 = interleukin-6, IL-12 = interleukin-12, IgG1 = immunoglobulin G1, IgG2 = immunoglobulin G2, IgM = immunoglobulin M, IFN-γ = interferon-γ, NS = not significant, TNF-α = tumor necrosis factor-α, WBC = white blood cells.

NK cell activity and cytokine levels were measured before (Visit 1: Week 0) and after (Visit 3: Week 8) administration. By comparing each measured value with that obtained at baseline (Visit 1), the degree of change between and within groups was determined. NK cell activity (primary outcome) tended to improve in the PL group compared with the placebo group after 8 weeks of intake (Fig. 2A; Table 2); at a ratio of 12.5:1, the PL group showed statistically significant improvement (5.62 ± 8.24) compared to the placebo group (0.95 ± 8.05) (*P* = .008) (Fig. 2B; Table 2). The WBC, TNF-α, IFN-γ, IL-1β, IL-2, IL-6, IL-12, and lgG1 (secondary outcomes) did not demonstrate significant improvement in the PL group versus the placebo group. In addition, the changes in the levels of IgG2 and IgM were observed but were not statistically significant (Fig. 3; Table 2).

**Table 2:** Comparison between placebo and PL group.

Variable	Visit 1 (Baseline)			Visit 3 (End of study)			Change from baseline				
	Placebo	PL	*P* value**	Placebo	PL	*P* value**	Placebo	*P* value*	PL	*P* value*	*P* value**
NK cell activity (50:1) (%)	49.77 (14.27)	47.92 (15.27)	.553	54.30 (13.06)	54.48 (13.75)	.948	4.53 (14.44)	.039	6.56 (12.08)	.001	.469
NK cell activity (25:1) (%)	35.95 (14.33)	34.33 (14.10)	.589	39.22 (13.45)	40.55 (12.69)	.629	3.27 (11.91)	.069	6.22 (11.35)	.001	.23
NK cell activity (12.5:1) (%)	22.20 (11.77)	19.80 (10.29)	.304	23.15 (10.68)	25.42 (9.04)	.276	0.95 (8.05)	.429	5.62 (8.24)	<.001	.008***
WBC (10^3^/mm^3^)	6.05 (1.19)	5.85 (1.45)	.459	5.50 (1.40)	5.29 (1.49)	.506	−0.56 (1.10)	.001	−0.55 (1.01)	.001	.986
TNF-α (pg/mL)	12.02 (4.42)	11.70 (3.27)	.697	10.85 (4.42)	11.49 (8.58)	.654	−1.18 (2.42)	.002	−0.21 (7.24)	.843	.396
IFN-γ (pg/mL)	41.45 (35.08)	38.48 (17.20)	.611	22.02 (25.34)	20.96 (8.60)	.79	−19.42 (15.30)	<.001	−17.52 (11.79)	<.001	.51
IL-1β (pg/mL)	2.80 (1.26)	3.14 (1.75)	.302	2.68 (1.18)	3.09 (1.51)	.154	−0.12 (0.71)	.246	−0.05 (0.82)	.703	.638
IL-2 (pg/mL)	4.29 (2.60)	4.53 (2.55)	.649	3.64 (2.29)	3.91 (1.95)	.552	−0.65 (0.96)	<.001	−0.63 (1.08)	<.001	.928
IL-6 (pg/mL)	7.46 (15.65)	5.34 (10.69)	.453	6.94 (14.90)	5.58 (12.09)	.634	−0.53 (2.16)	.105	0.24 (2.36)	.507	.112
IL-12 (pg/mL)	5.53 (2.77)	5.40 (1.94)	.797	4.76 (2.55)	5.01 (2.08)	.607	−0.77 (1.44)	.001	−0.39 (1.18)	.032	.172
IgG1 (mg/dL)	614.06 (135.35)	641.27 (132.15)	.335	597.96 (132.37)	632.94 (136.69)	.218	−16.10 (44.76)	.019	−8.33 (57.44)	.336	.473
IgG2 (mg/dL)	453.25 (133.87)	463.23 (131.04)	.72	457.16 (139.07)	455.15 (123.73)	.942	3.91 (34.75)	.449	−8.08 (29.81)	.076	.081
IgM (mg/dL)	129.66 (61.64)	125.31 (52.36)	.718	128.67 (61.56)	122.19 (50.12)	.584	−0.99 (10.87)	.539	−3.12 (7.64)	.009	.285

Data are presented as mean (standard deviation).

IL-1β = interleukin-1β, IL-2 = interleukin-2, IL-6 = interleukin-6, IL-12 = interleukin-12, IgG1 = immunoglobulin G1, IgG2 = immunoglobulin G2, IgM = immunoglobulin M, IFN-γ = interferon-γ, NK = natural killer, PL = *Phellinus linteus*, TNF-α = tumor necrosis factor-α, WBC = white blood cells.

* *P* values were compared within each group (paired *t* test).

** *P* values were compared between groups (independent *t* test).

*** *P* values was compared between groups (independent *t* test).

Safety was evaluated through adverse event recording and clinical laboratory testing in the intention-to-treat group. A total of 29 adverse events were reported in 23 subjects (23.5%) (Table 3). Adverse reactions were reported in 11 subjects (16 cases) in the PL group and 12 subjects (13 cases) in the placebo group. All adverse reactions were not originating from the intake of PL extract, and there were no occasions of breaking the code. In this study, there was no dropout of study participants due to adverse events. One serious adverse event (such as hospitalization due to a sprain in the lumbar spine), was not associated with the intervention, and such case was reported from just 1 subject (1%) (Table 3). The follow-up evaluation revealed that this serious adverse reaction resolved spontaneously, and that the subject dropped out due to the withdrawal of consent after discharge. The results of clinical laboratory tests were compared between groups and against baseline measurements. In the safety analysis, there was no significant difference in changes observed between groups (Table 4). Moreover, there were no specific adverse reactions or abnormalities in terms of clinical implications or physical examinations.

**Table 3:** Adverse events.

Complaints	Severity*	Cause-and-effect relationship†	Group (randomization number)
Upper respiratory tract infection	A	5	Placebo (013, 061), PL (022, 065)
Cervicalgia	A	5	Placebo (031, 076), PL (037, 045, 055)
Elevated blood glucose levels	A	5	Placebo (054), PL (041)
Indigestion	A	4	Placebo (044, 098), PL (096)
	A	5	Placebo (040)
Increased WBC	A	4	Placebo (006)
Decreased WBC	A	4	PL (085)
Elevated blood ALT levels	A	5	Placebo (021)
Diarrhea and stomach pain	A	5	PL (032)
Shoulder sprain due to TA	A	5	PL (029)
Lumbar sprain due to TA	A	5	PL (029)
Lumbago	A	5	Placebo (076), PL (032)
Lumbar sprain	B	5	PL (032)
Diarrhea	A	5	PL (033)
Insomnia	A	5	PL (037)
Wrist pain	A	5	Placebo (081)
Stomach pain	A	5	PL (022)
Constipation	A	5	Placebo (051)
Lumbar sprain (serious adverse event)	B	5	PL (032)

ALT = alanine aminotransferase, PL = *Phellinus linteus*, TA = traffic accident, WBC = white blood cell.

* A, mild; B, moderate; C, severe.

† 1, clearly related; 2, thought to be related; 3, likely to be related; 4, thought to be unrelated; 5, thought to be clearly unrelated; 6, unidentified.

**Table 4:** Laboratory test results in ITT population.

Variable	Observed value			Change from baseline				
	Placebo	PL	*P* value*	Placebo	*P* value*	PL	*P* value*	*P* value**
RBC (10^6^/mm^3^)								
Screening	4.43 (0.41)	4.41 (0.35)	.818					
Visit 3	4.36 (0.44)	4.37 (0.37)	.98	−0.06 (0.17)	0.015	−0.04 (0.19)	.129	.594
Hb (g/dL)								
Screening	13.66 (1.31)	13.27 (1.56)	.186					
Visit 3	13.36 (1.49)	13.13 (1.35)	.436	−0.30 (0.55)	<.001	−0.14 (0.67)	.162	.191
Hct (%)								
Screening	40.27 (3.05)	39.28 (3.71)	.153					
Visit 3	39.67 (3.59)	39.13 (3.24)	.437	−0.60 (1.56)	.01	−0.15 (1.93)	.596	.206
Platelet (10^3^/mm^3^)								
Screening	256.92 (49.10)	263.57 (60.07)	.55					
Visit 3	247.65 (47.34)	256.67 (60.80)	.415	−9.27 (19.52)	.002	−6.90 (29.97)	.114	.644
Na (mmol/L)								
Screening	140.37 (1.82)	140.63 (1.69)	.457					
Visit 3	140.31 (1.86)	140.22 (1.49)	.811	−0.06 (1.63)	.793	−0.41 (1.47)	.058	.271
K (mmol/L)								
Screening	4.41 (0.39)	4.41 (0.36)	.957					
Visit 3	4.32 (0.41)	4.34 (0.36)	.754	−0.09 (0.33)	.057	−0.06 (0.46)	.338	.724
Cl (mmol/L)								
Screening	102.14 (2.04)	102.55 (2.04)	.325					
Visit 3	103.61 (2.43)	103.57 (1.89)	.926	1.47 (2.53)	<.001	1.02 (2.46)	.006	.375
Triglyceride (mg/dL)								
Screening	116.00 (53.78)	138.06 (107.51)	.203					
Visit 3	119.96 (62.11)	129.41 (87.57)	.539	3.96 (42.88)	.521	−8.65 (86.91)	.489	.365
Total cholesterol (mg/dL)								
Screening	203.57 (36.48)	206.18 (34.79)	.718					
Visit 3	205.53 (38.54)	199.47 (36.62)	.427	1.96 (26.34)	.605	−6.71 (22.28)	.04	.082
AST (U/L)								
Screening	25.86 (9.09)	27.24 (13.13)	.544					
Visit 3	26.33 (12.87)	24.55 (8.73)	.426	0.47 (12.98)	.801	−2.69 (11.60)	.111	.207
ALT (U/L)								
Screening	23.98 (19.77)	23.69 (19.52)	.943					
Visit 3	26.31 (26.43)	22.08 (16.18)	.342	2.33 (26.00)	.534	−1.61 (9.30)	.231	.321
ALP (U/L)								
Screening	63.73 (15.41)	67.57 (19.89)	.288					
Visit 3	63.29 (14.12)	65.61 (20.28)	.512	−0.45 (6.99)	.655	−1.96 (8.10)	.097	.325
γ-GTP (U/L)								
Screening	19.98 (13.77)	26.65 (26.09)	.118					
Visit 3	20.33 (17.21)	28.04 (33.16)	.153	0.35 (12.63)	.848	1.39 (24.34)	.692	.791
Total bilirubin (mg/dL)								
Screening	0.72 (0.28)	0.75 (0.29)	.621					
Visit 3	0.70 (0.25)	0.73 (0.31)	.706	−0.02 (0.20)	.604	−0.02 (0.21)	.467	.871
Glucose (mg/dL)								
Screening	97.37 (8.67)	96.27 (8.08)	.517					
Visit 3	98.96 (10.78)	97.84 (10.98)	.611	1.59 (7.59)	.148	1.57 (9.62)	.259	.991
BUN (mg/dL)								
Screening	13.58 (3.55)	14.16 (3.52)	.414					
Visit 3	14.93 (4.26)	14.99 (3.93)	.943	1.35 (3.31)	.006	0.82 (3.15)	.074	.422
Creatinine (mg/dL)								
Screening	0.70 (0.16)	0.69 (0.14)	.867					
Visit 3	0.69 (0.16)	0.68 (0.14)	.669	−0.01 (0.07)	.472	−0.01 (0.05)	.059	.509
ESR (mm/h)								
Screening	12.06 (7.65)	14.31 (9.66)	.205					
Visit 3	14.65 (10.51)	15.31 (10.65)	.761	2.59 (7.80)	.024	1.00 (7.89)	.380	.318

Data are presented as mean (standard deviation).

ALP = alkaline phosphatase, ALT = alanine transaminase, AST = aspartate aminotransferase, BUN = blood urea nitrogen, ESR = erythrocyte sedimentation rate, Hb = hemoglobin, Hct = hematocrit, ITT = intention-to-treat, PL = *Phellinus linteus*, RBC = red blood cell, γ-GTP = γ-glutamyl transpeptidase.

* *P* values were compared within each group (paired *t* test).

** *P* values were compared between groups (independent *t* test).

## 4. Discussion

Immunity recognizes, metabolizes, and removes external antigens that invade the human body. Therefore, maintaining the homeostasis of immunity is important. Immune hyperactivity causes various autoimmune disorders, allergic rhinitis, hay fever, allergic asthma, atopic dermatitis, rheumatoid arthritis, and psoriasis. On the other hand, impaired immunity reduces the defensive capability of an organism and renders individuals vulnerability to infection.^[11]^ WBCs that play a key role in immunity are differentiated from the stem cells. WBCs are subcategorized into granulocytes, monocytes, and lymphocytes, which can be further divided into T, B, and NK cells.^[12]^ NK cells defend their hosts by attacking cancer cells or virus-infected cells.^[13]^ NK cells are selectively cytotoxic to cancer cells through various immune receptors present on the cell surface. NK cells recognize the target cells and are activated through germ-line encoded immune receptor.^[14]^ Representatively, the natural killer group 2 member D (NKG2D) receptor in NK cells detects UL16 binding proteins (ULBPs) and MICA/B, which are increased when DNA is damaged, or in cases of cancer and viral infection.^[15]^ NK cells also recognize the deficiency of MHC Class I and remove the missing self.^[16]^ In addition, NK cells interact with dendritic cells, macrophages, and T cells and regulates inflammatory reactions through the production of cytokines such as IFN-γ or TNF-α. In particular, cancer stem cells are effectively removed by NK cells.^[17]^ Cancer stem cells are highly resistant to anticancer drugs and radiation therapy, which cause cancer stem cells to recur even after remission.^[18]^ Decrease in NK cell activity means an environment in which cancer cells can grow. They are responsible for innate immunity and any change in NK cell activity can serve as a biomarker for predicting immunity disorders (e.g., cancer occurrence and metastasis, viral infection).^[19,20]^

Immune enhancers have been extensively investigated throughout the plant kingdom.^[21]^ Studies on polysaccharides contained in mushrooms have been conducted since the 1950s. Protein-bound polysaccharides isolated from basidiomycetes exhibit anticancer effects through the host’s immunity.^[22]^ Notably, polysaccharides obtained from fungi have also exhibited antitumor and immunomodulatory activities.^[23]^ Most of these protein-bound polysaccharides are beta-glucan systems, which are widely used for their immune-strengthening and anticancer effects.^[24]^ Extensive research has shown that polysaccharides extracted from mushrooms are safe. Recently, Lentinan and Meshima derived from mushrooms have been utilized as one of the prescription medicines in anticancer treatment and immunotherapy.

PL belongs to the *Hymenochaetaceae* family, of which PL and *Phellinus igniarius* are the most well-established types.^[25]^ PL has shown excellent anticancer and immunity-enhancing effects. This clinical trial confirmed the potential use of PL extract as one of the functional ingredients to improve the immunity. The efficacy and safety of PL were previously demonstrated in a nonclinical trial. In addition, a pilot clinical trial revealed that treatment with PL extract increased the activity of NK cells. The PL and placebo group were homogeneous based on the lack of differences in the basic characteristics of the subjects (Table 1).

The primary outcome of NK cell activity was measured using the LDH cytotoxicity assay. This method isolates NK cells from the blood, and allows NK cells to react with K562 cells (chronic myelogenous leukemia cells) in various ratios (50:1, 25:1, and 12.5:1), and measures the concentration of LDH released after cell dissolution is determined. This method is used to measure NK cell activity.^[26]^ The amount of LDH released in the PL group (12.5:1, effector cell: target cell) was significantly improved in comparison with LDH levels measured in the placebo group (*P* = .008) (Fig. 2B). The secondary outcomes (WBC, TNF-α, IFN-γ, IL-1β, IL-2, IL-6, IL-12, IgG1, IgG2, and IgM) did not demonstrate significant changes; however, they showed a tendency for improvement that may contribute to enhanced immunity (Fig. 3). Cytokines are greatly influenced by the individual characteristics, such as the diversity of peripheral monocytes, nongenetic factors (e.g., age, weight, and season), balance of intestinal microorganisms, and diversity of the microbiome due to differences in lifestyle.^[27]^ In addition, it has been reported that seasonal variations influence the levels of IFN-γ and IL-10 (i.e., increased in summer and autumn and decreased in winter).^[28]^ This 8-week clinical trial was conducted during autumn/winter, and the reduction in cytokines was considered in its physiological context. In future studies, to compensate for such variations, it is necessary to consider cytokine secretion depending on the season in which the clinical trial was performed, and conduct statistical analysis excluding the outliers to control variations originating from the individual characteristics.

These results suggest that the intake of PL mycelium extract improves the immune function of individuals. In addition, the safety evaluation, through adverse event recording, laboratory testing, and vital sign monitoring, confirmed that the PL extract is not associated with hepatotoxicity and renal toxicity (Table 4). Therefore, the long-term consumption of PL extract is considered safe. In order to evaluate the therapeutic benefits of consuming the PL extract, further investigation should be carried out with patients with compromised immunity, in addition to the healthy individuals tested in this clinical trial.

## 5. Conclusion

This clinical trial evaluated the efficacy and safety of PL extract to be used as one of the major functional ingredients in nutritional supplement. NK cell activity was significantly improved in the concentration of 12.5:1 (effector cell:target cell) in the PL group compared with the placebo group. The level of WBC, TNF-α, IFN-γ, IL-1β, IL-2, IL-6, IL-12, and IgG1 also improved upon intake of PL extract. Moreover, there were no PL extract-related side effects observed in terms of adverse reactions, clinical laboratory test results, vital signs, and physical examination during the clinical trial. The results of this clinical trial suggest that the oral administration of PL extract for 8 weeks is safe and may enhance performances of individual immune system.

## Author contributions

All authors participated in the clinical trial and reviewed the manuscript. YHK and JHK acquired the data and performed the analysis. JHK and HL supervised the clinical trial edited the article.

**Conceptualization:** Jae Hui Kang, Hyun Lee.

**Data curation:** Yong Ho Ku, Jae Hui Kang.

**Formal analysis:** Yong Ho Ku.

**Funding acquisition:** Jae Hui Kang.

**Investigation:** Jae Hui Kang.

**Resources:** Yong Ho Ku, Jae Hui Kang, Hyun Lee.

**Supervision:** Jae Hui Kang, Hyun Lee.

**Visualization:** Yong Ho Ku, Jae Hui Kang, Hyun Lee.

**Writing – original draft:** Yong Ho Ku.

**Writing – review & editing:** Jae Hui Kang, Hyun Lee.
